# Immuno-Expression of Endoglin and Smooth Muscle Actin in the Vessels of Brain Metastases. Is There a Rational for Anti-Angiogenic Therapy?

**DOI:** 10.3390/ijms15045663

**Published:** 2014-04-02

**Authors:** Valeria Barresi, Giovanni Branca, Maria Caffo, Rosario Caltabiano, Antonio Ieni, Enrica Vitarelli, Salvatore Lanzafame, Giovanni Tuccari

**Affiliations:** 1 Department of Human Pathology “G. Barresi”, University of Messina, Messina 98125, Italy; E-Mails: gianni_branca@alice.it (G.B.); calaienco@hotmail.com (A.I.); evitarelli@unime.it (E.V.); tuccari@unime.it (G.T.); 2 Department of Neurosciences, University of Messina, Messina 98125, Italy; E-Mail: mcaffo@unime.itm; 3 Department G.F. Ingrassia, Section of Anatomic Pathology, University of Catania, Catania 95123, Italy; E-Mails: rosario.caltabiano@unict.it (R.C.); lanzafas@unict.it (S.L.)

**Keywords:** brain metastasis, endoglin, MVD, pericytes, angiogenesis

## Abstract

Despite ongoing clinical trials, the efficacy of anti-angiogenic drugs for the treatment of brain metastases (BM) is still questionable. The lower response rate to anti-angiogenic therapy in the presence of BM than in metastatic disease involving other sites suggests that BM may be insensitive to these drugs, although the biological reasons underlining this phenomenon are still to be clarified. With the aim of assessing whether the targets of anti-angiogenic therapies are actually present in BM, in the present study, we analyzed the microvessel density (MVD), a measure of neo-angiogenesis, and the vascular phenotype (mature *vs.* immature) in the tumor tissue of a series of BM derived from different primary tumors. By using immunohistochemistry against endoglin, a specific marker for newly formed vessels, we found that neo-angiogenesis widely varies in BM depending on the site of the primary tumor, as well as on its histotype. According to our results, BM from lung cancer displayed the highest MVD counts, while those from renal carcinoma had the lowest. Then, among BM from lung cancer, those from large cell and adenocarcinoma histotypes had significantly higher MVD counts than those originating from squamous cell carcinoma (*p* = 0.0043; *p* = 0.0063). Of note, MVD counts were inversely correlated with the maturation index of the endoglin-stained vessels, reflected by the coverage of smooth muscle actin (SMA) positive pericytes (*r* = −0.693; *p* < 0.0001). Accordingly, all the endoglin-positive vessels in BM from pulmonary squamous cell carcinoma and renal carcinoma, displayed a mature phenotype, while vessels with an immature phenotype were found in highly vascularized BM from pulmonary large cell and adenocarcinoma. The low MVD and mature phenotype observed in BM from some primary tumors may account for their low sensitivity to anti-angiogenic therapies. Although our findings need to be validated in correlative studies with a clinical response, this should be taken into account in therapeutic protocols in order to avoid the adverse effects of useless therapies.

## 1. Introduction

Metastatic brain tumors represent the most common intracranial neoplasm in the adult [1] and a significant cause of mortality and morbidity in patients suffering from cancer [2]. An increase in their frequency has been noted in the last few years, presumably depending on the longer overall survival of cancer patients, on the widespread use of brain magnetic resonance imaging (MRI) in the upfront staging and follow-up, as well as on the use of novel therapeutic compounds with good anti-neoplastic activity, but inadequate penetration via the blood-brain barrier [3]. Although their exact incidence is unknown, it has been estimated that brain metastases (BM) can occur in up to 30% of patients affected by various types of cancer [4]. The rate of occurrence of brain metastatic tumors is strongly correlated to the tumor type, with lung and breast carcinomas as the most common sources, followed by melanoma, renal and gastro-intestinal malignancies [5]. It has been shown that the metastatic potential of a tumor is strictly linked to its ability to induce neo-angiogenesis, *i.e.*, the formation of new blood vessels from pre-existing ones. Indeed, the newly formed vessels inside the tumor provide an entry site into the circulation for the neoplastic cells that detach from the tumor mass [6]; then, these may spread, arrest in the capillary beds of distant organs, extravasate, proliferate and produce a successful metastasis [6–8]. Furthermore, the growth of the secondary tumor may depend upon the potential of the cancer cells to induce neo-angiogenesis at the metastatic site. Indeed, tumors that show more intense angiogenesis produce large, fast-growing parenchymal BM, while tumors characterized by less intense angiogenesis give rise to few, slow growing BM [8]. On this evidence, novel therapeutic strategies impairing the neo-angiogenic process have been developed in order to reduce the growth and risk of the progression of malignant tumors. For instance, recombinant humanized monoclonal antibodies have been employed to neutralize the biological activity of several human angiogenic factors, among which vascular endothelial growth factor (VEGF) is the most powerful [9]. On the evidence that VEGF promotes the formation of BM in animal models, while its inhibition results in attenuated BM growth [10], this factor was proposed as a target for the treatment of BM from human cancer. Although anti-angiogenic therapies have been used in this indication only in recent times [11], conflicting findings have been reported on their efficacy in BM [12–18].

The inefficacy of anti-angiogenic drugs may find an explanation in the independency of BM growth from neo-angiogenesis [19]. In addition, the phenotype of the tumor vessels in BM may influence responsiveness to these treatments. Indeed, it is known that during angiogenesis, immature vessels, devoid of a pericyte covering, are firstly formed [20]; then, when maturation occurs (so-called normalization), vessels acquire a pericyte coating [21]. Since only immature tumor vessels are VEGF-dependent for survival [21] and tumor vessels resistant to anti-angiogenic treatments display pericyte coverage [22], a high maturation index of vessels in BM may account for the insensitivity to these drugs.

With the aim of providing an explanation for which anti-angiogenic drugs may not work on BM, in the present study, we analyzed the effective presence of their targets in tumor tissue from BM. Thus, we assessed the number of newly formed vessels and their pericyte coating in a series of BM submitted to surgical resection. The presence of pericytes was investigated by using immunohistochemistry against α-smooth muscle actin (SMA), a marker of mature pericytes, while neo-angiogenesis was quantified by assessing the so-called microvessel density (MVD), which reflects the number of vessels/mm^2^ [23], through standard immunohistochemistry against endoglin (CD105). This is a 180-kDa transmembrane homodimeric glycoprotein that belongs to the TGFβ receptor complex [24] and which has been demonstrated to be a more specific marker for neo-angiogenesis in comparison to pan-endothelial markers [23].

## 2. Results

The clinico-pathological characteristics, as well as the immunohistochemical findings relative to the tumors in the study are summarized in Table 1.

**Figure 1.**
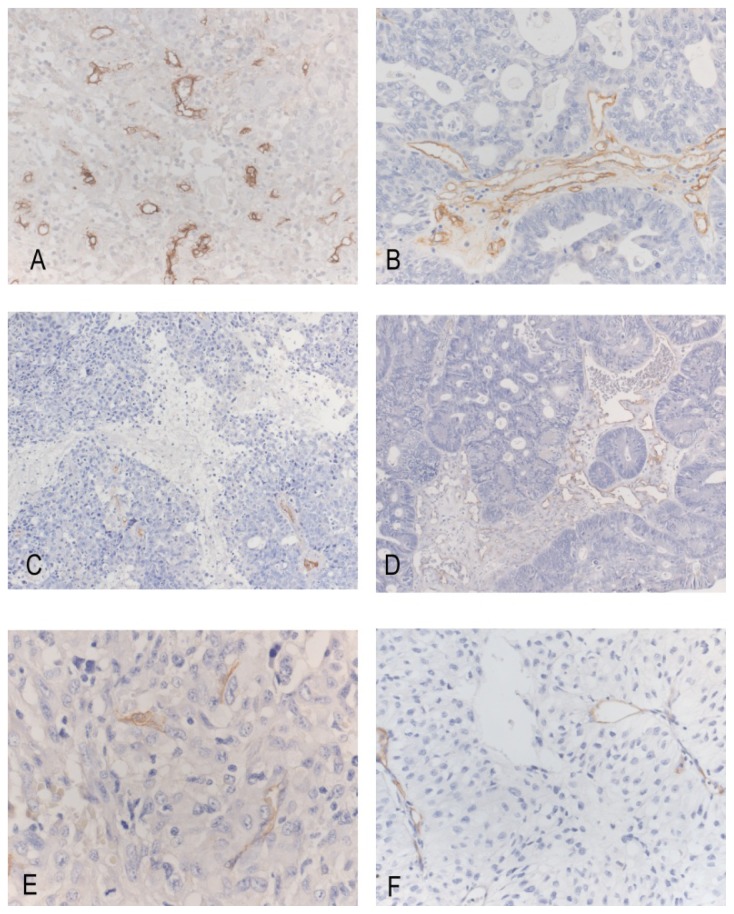
Endoglin-positive vessels in BM from (**A**) large cell carcinoma (endoglin stain; original magnification; ×200); (**B**) adenocarcinoma of the lung (endoglin stain; original magnification; ×200); (**C**) breast ductal adenocarcinoma (endoglin stain; original magnification; ×200); (**D**) CRC (endoglin stain; original magnification; ×100); (**E**) pulmonary squamous cell carcinoma (endoglin stain; original magnification; ×200); and (**F**) CCRCC (endoglin stain; original magnification; ×200).

**Figure 2.**
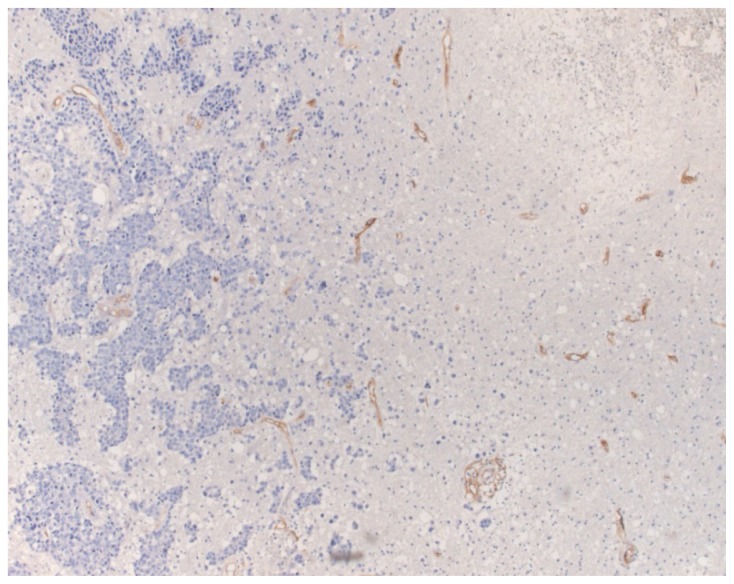
Endoglin-stained vessels in the brain parenchyma adjacent to a BM from pulmonary large cell carcinoma (endoglin stain; original magnification; ×100).

Endoglin immunostained microvessels were evidenced in all of the analyzed metastases, as well as in the brain parenchyma surrounding the tumor (Figures 1 and 2). The MVD ranged between 1.11 and 140 vessels/mm^2^ within the tumor mass. When the primary tumor was considered, MVD ranged between 6 and 140 (median MVD: 56.6; mean MVD: 58.85 ± 33.84) in BM from lung carcinomas, between 1.11 and 90 (median MVD: 36; mean MVD: 39.43 ± 28.17) in BM from breast cancer, between 2.22 and 57.3 (median MVD: 20.52; mean MVD: 25.84 ± 15.1) in those from melanoma, between 10 and 93.3 (median value: 32.7; mean MVD: 42.17 ± 37.48) in those from CRC carcinoma, between 1.11 and 40 (median MVD: 21.65; mean MVD: 21.10 ± 15.94) in those from CCRCC, between 11.3 and 93.3 (median MVD: 40; mean MVD: 39.98 ± 33.38) in metastatic papillary serous ovarian carcinoma and between 4 and 113.3 (median MVD: 9.3; mean MVD: 42.2 ± 61.63) in the metastases from uterine carcinoma (Table 2) (Figure 1).

BM from lung, large bowel and uterine cancers were from tumors of different histotypes. In detail, among the 39 metastases from pulmonary carcinoma, 27 were from adenocarcinomas, 6 from squamous carcinomas, 5 from large cell carcinomas and 1 from small cell carcinoma. The metastases from large cell carcinomas displayed the highest MVD counts, which ranged between 66.6 and 123.3 (median MVD: 83.3; mean MVD: 93.16 ± 23.12) (Figure 1A), followed by those from adenocarcinomas (Figure 1B), with an MVD ranging between six and 140 (median MVD: 56.6; mean MVD: 58.7 ± 31.49), and those from squamous cell carcinomas (Figure 1E) featuring MVD counts comprised between 12 and 53.3 (median MVD: 21.4; mean MVD: 24.48 ± 15.05) (Table 2). Among the 4 metastases from CRC, two displayed a mucinous histotype and were characterized by lower MVD counts. When we considered the metastases from uterine carcinoma, that from endometrioid carcinoma had the highest MVD count (MVD: 113.3), while only sparse newly formed vessels were evidenced in the metastases from clear cell adenocarcinoma and serous carcinoma (Table 2).

The Mann–Whitney test revealed that BM from lung cancer had significantly higher MVD counts than those from melanoma (*p* = 0.0096) or CCRCC (*p* = 0.024); no other statistically significant differences in the MVD counts between the different tumor types were found.

When the BM from lung cancer were subdivided according to the histotype, those from large cell carcinomas displayed significantly higher MVD counts compared to those from adenocarcinomas or squamous cell carcinomas (*p* = 0.0293; *p* = 0.0043) (Table 2). In addition, brain metastatic tumors from pulmonary large cell carcinoma had significantly higher MVD than those from breast cancer, melanoma or CCRCC (*p* = 0.0044; *p* = 0.0016; *p* = 0.0159) (Table 2). Again, BM from lung adenocarcinoma had significantly higher MVD than those from squamous cell lung carcinomas, melanomas or CCRCC (*p* = 0.0063; *p* = 0.008; *p* = 0.0157) (Table 2). No significant correlation was found between the MVD of the tumors and their mean diameter by the Spearman correlation test (*r* = 0.0668; *p* = 0.5612; 95% CI: −0.158–0.285).

**Figure 3.**
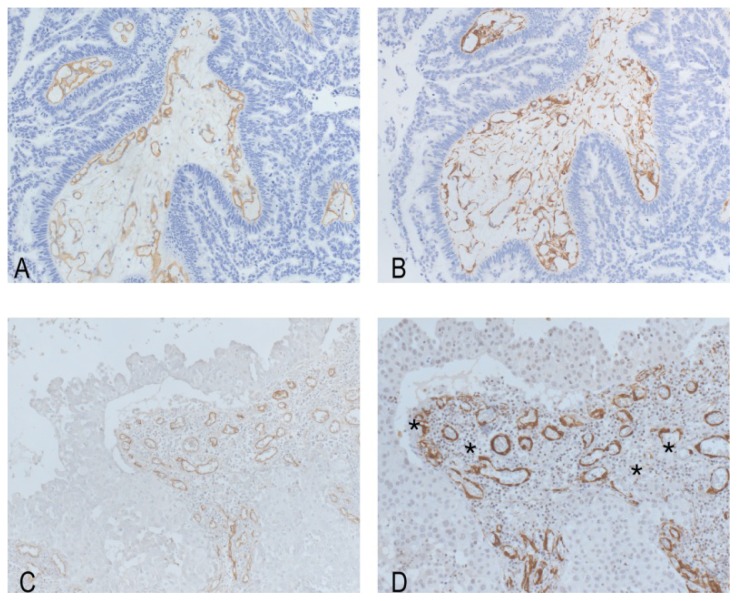
Endoglin-stained vessels in a case of BM from serous papillary ovarian carcinoma (**A**) (endoglin stain; original magnification; ×200) and in a BM from lung adenocarcinoma (**C**) (endoglin stain; original magnification; ×200). A consecutive section showing that endoglin-positive vessels were also stained by anti-smooth muscle actin (SMA) antibody in BM from ovarian carcinoma (**B**) (endoglin stain; original magnification; ×200); while SMA stain was incomplete in some of the endoglin-positive vessels in BM from lung adenocarcinoma (**D**) (SMA stain; original magnification; ×200). The stars indicate vessels with incomplete pericyte coating.

In the BM of our series, the maturation index of endoglin-stained vessels ranged between 52% and 100% (Figure 3), with most of the cases (66%) showing an index of 100%. Interestingly, an index of 100% was evidenced in all of the metastases from CCRCC, melanoma, ovarian serous papillary carcinomas, CRC, thymic and pulmonary squamous cell carcinomas, as well as in MPNST (Table 1). Even more, a significant inverse correlation was found between MVD counts and the maturation index (*r* = −0.693; *p* < 0.0001). Indeed, BM from lung large cell carcinomas had significantly lower maturation fractions compared to those from pulmonary squamous cell carcinoma, ovarian carcinoma, melanoma, CCRCC and CRC (*p* = 0.0041; *p* = 0.0071; *p* = 0.0015; *p* = 0.0127; *p* = 0.0127) (Table 2). Endoglin-positive vessels did not differ in their diameter on the basis of their maturation status (*p* = 0.3404).

A trend towards correlation was evidenced between lower overall survival of the patients and higher MVD (MVD > 56.6) (*p* = 0.0501) or a lower maturation fraction of endoglin-stained vessels (index < 1) (*p* = 0.0859) in the BM, though statistical significance was not reached.

## 3. Discussion

Following the evidence that neo-angiogenesis is essential for tumor growth [25], a series of treatments aimed at interfering with this process have been developed [26,27].

Despite the multiple clinical trials [10–18,26–29], conflicting findings have been reported on the role of anti-angiogenic drugs in the treatment of BM [10–18,26–29], and definite confirmation of their effectiveness in this indication is still lacking. Besides, the lower response rate to anti-angiogenic treatment in the presence of BM than in metastatic disease involving other sites suggests that BM may be insensitive to these drugs [18].

At present, no valid biomarkers able to predict the efficacy of anti-angiogenic therapies in BM have been identified. Efforts to find predictive markers have mainly focused on available tissue samples from primary tumor resections [30,31], to make inferences concerning the treatment of metastatic disease. Nonetheless, a recent study on non-small cell lung cancer [32] showed that the vascular phenotype in BM is different from that of primary tumors, suggesting that biomarkers predictive of response should be better evaluated on the target tissue.

Given that little is known on the biology of angiogenesis in BM [31,32], in the present study, we analyzed the density and the phenotype (mature *vs*. immature) of newly formed vessels in tissue derived from a series of brain metastatic tumors; indeed, we believe that having more information on the vascular phenotype of BM may help to understand whether there is a rationale in the use of antiangiogenic agents in this indication.

In the BM of our series, MVD was investigated by using immunohistochemistry against endoglin, a protein that is predominantly expressed on cycling vascular endothelial cells in regenerating inflamed or neoplastic tissues [33] and only weakly, or not expressed at all, in the vascular endothelium of normal tissues [23]. For this reason, endoglin appears to be more specific in the detection of intra-tumoral neo-angiogenesis in comparison to pan-endothelial markers, such as CD34 and CD31, which also react with pre-existing normal host vessels.

We found a wide variation in the MVD counts of BM included in our series, with values ranging between 1.11 and 140 vessels/mm^2^. Since MVD assessed by endoglin immunostain represents a measure of neo-angiogenesis, this suggests that BM may be more or less dependent on neo-angiogenesis for their growth, and as a consequence, they may show different responsiveness to anti-angiogenic drugs. Although for many years, it was believed that neo-angiogenesis was the sole mechanism able to provide vascular supply to a tumor, it has been demonstrated that tumors may be vascularized through alternative processes [34], such as vasculogenic mimicry [35] or vascular co-option [22,36]. These mechanisms may ensure tumor supply independently of neo-angiogenesis. Indeed, in vasculogenic mimicry, highly aggressive tumor cells form vessel-like structures by virtue of their high plasticity, with no involvement of endothelial cells [35]. Thus, the target of treatment, endothelial cells, does not exist, making conventional antiangiogenic therapy ineffective. In addition, due to the high intrinsic vascular density of brain, tumors at this site may grow regardless of the presence of angiogenesis by using the pre-existing vessels (so called co-option [37–39]), which may also account for resistance to anti-angiogenic drugs.

*In vitro* studies or clinical trials [8,18,40] indicate that BM have a different response to angiogenic treatments according to the site of the primary tumor and to its histotype. Interestingly, when BM of our series were subdivided according to their origin, those from pulmonary carcinomas displayed the highest MVD counts, while lower counts were evidenced in those from breast cancer, melanoma, CCRCC or colorectal, uterine and ovarian cancers, though statistical significance was reached only with regards to melanoma and CCRCC. Similar findings had been previously achieved by Salgado and co-workers, who had also investigated endoglin expression in the vessels of BM [32] and had found less intense angiogenesis in BM from melanoma in comparison to those from lung and breast cancer. Nevertheless, BM from breast carcinomas were the most vascularized in their study [32]. This discrepancy compared to our findings might be justified by the prevalence of BM from lung cancer with the squamous cell histotype in their series [32]. Indeed, in our BM, the MVD counts did not differ only according to their primitivity, but also the histotype, and those from the squamous cell carcinoma of the lung had a significantly lower number of endoglin-positive vessels in comparison to those from large cell carcinomas and adenocarcinomas. Furthermore, among BM from CRC, those with a mucinous histotype had lower MVD counts, and a remarkable difference existed between the highly vascularized endometrioid histotype and clear cell or serous variants, which showed a low number of newly formed vessels. In our study, also the pericyte coating of endoglin-stained vessels differed according to the origin and histotype of BM. The maturation fraction of the vessels was 100% in all BM, with the exception of those derived from lung, breast cancer or uterine endometrioid carcinoma. This means that newly formed vessels in brain metastatic tumors from melanoma, CCRCC, colorectal, clear cell and serous uterine carcinomas and ovarian cancer showed a mature phenotype, while some BM from pulmonary large cell carcinoma or adenocarcinoma and from breast carcinoma had immature vessels.

Our findings diverge from those reported in primary lung tumors, where the average number of endoglin-positive vessels does not show any significant differences between adenocarcinoma and squamous cell carcinoma of the lung [41] and the degrees of maturation in newly formed blood vessels are less developed in the inner areas of squamous cell carcinoma than adenocarcinoma [41]. Nevertheless, a recent study showed that neither the VEGF expression nor the MVD or vascular phenotype can be predicted in the brain metastasis from an examination of the primary lung carcinoma. In addition, experimental studies confirm that BM with a different origin may have different angiogenic and growth potential, as lung adenocarcinoma and colon carcinoma cells give rise to large, fast-growing parenchymal brain metastases, whereas lung squamous cell carcinoma, renal cell carcinoma and melanoma cells produce only a few slow-growing brain metastases [8].

Since only immature (pericyte coverage devoid) vessels seem to be responsive to VEGF action [22] and tumor vessels resistant to anti-angiogenic treatments display pericyte coverage [22], we may speculate that sensitivity to anti-angiogenic therapies may differ in tumors with different origins and histotypes, due to their different proportion of vessels with an immature phenotype. The lower angiogenic potential and higher maturation fraction in BM from melanoma than in those from lung cancer may explain why the VEGF-A inhibitor, bevacizumab, blocks angiogenesis and results in the dormancy of the former, but not of the latter [36]. In addition, our results may clarify why VEGF blockage decreases the incidence of BM from lung adenocarcinoma, but not that of BM from lung squamous cell carcinoma [8]. Similarly, the failure of anti-angiogenic therapy in CCRCC metastatic to the brain [42] may depend upon low MVD and the presence of pericyte coverage in BM from this tumor.

## 4. Materials and Methods

All the procedures were performed in compliance with relevant laws and institutional guidelines and approved by the local institutional committee of Policlinic G. Martino, Messina, Italy.

Following the revision of our departmental databases, we retrospectively selected and included in the study 78 BM, which had been surgically treated in our institutions between 2006 and 2013. None of the patients had received neo-adjuvant therapy for their brain tumor. In the selection process, we considered only those cases in which the primary tumor had been identified. In detail, the primary tumor was pulmonary carcinoma in 39 cases, breast carcinomas in 13, ovarian carcinoma in 5, melanoma in 8, colorectal carcinoma (CRC) in 4, clear cell renal cell carcinoma (CCRCC) in 4, uterine carcinoma in 3, thymic carcinoma in 1 and malignant peripheral nerve sheath tumor (MPNST) in 1.

The paraffin blocks and corresponding histological slides were available for each brain metastatic tumor. Hematoxylin and eosin slides were preliminarily reviewed in order to establish the eventual histotype of the tumor and to select the paraffin block with the least amount of necrosis and the highest amount of neoplastic tissue for the immunohistochemical analyses.

### Immunohistochemistry, Quantification and Statistics

Four micrometer-thick consecutive sections were cut from the paraffin blocks of the BM and submitted to the immunohistochemical procedures against endoglin and SMA. Briefly, the endogenous peroxidase activity was preliminary blocked with 0.1% H_2_O_2_ in methanol for 20 min; then, normal sheep serum was applied for 30 min to prevent the unspecific adherence of serum proteins. For the endoglin epitope retrieval, specimens were pre-treated with proteinase K (S3020, DAKO Cytomation, Glostrup, Denmark) at room temperature for 15 min, while the SMA antigen was unmasked by microwave oven pre-treatment in 10 mM, pH 6.0 sodium citrate buffer for 3 cycles × 5 min. Sections were successively incubated with the primary monoclonal antibodies against endoglin (DAKO Cytomation, Glostrup, Denmark, clone SN6h, working dilution, 1:50) and SMA (DAKO Corporation, Denmark, clone 1A4, working dilution, 1:100). The bound primary antibodies were visualized by using the LSAB kit (DAKO Cytomation, Glostrup, Denmark) according to the manufacturer’s instructions. To reveal the immunostaining, the sections were incubated in darkness for 10 min with 3′–3′ diaminobenzidine tetra hydrochloride (Sigma Chemical Co., St. Louis, MO, USA), in the amount of 100 mg in 200 mL of 0.03% hydrogen peroxide in phosphate-buffered saline (PBS) solution. Nuclear counterstaining was performed by Mayer’s haemalum (Diapath S.p.a., Bergamo, Italy). The specificity of the binding was assessed by omitting the primary antiserum or replacing it with normal rabbit serum or phosphate buffered saline solution (PBS, pH 7.4). Moreover, renal tubules within specimens of fetal kidney and the syncytiotrophoblast present in specimens of human term placenta were used as the positive control for endoglin immuno-reaction [23].

The assessment of MVD was performed within the cancerous mass, excluding all the necrotic areas, as previously described [23,43–51]. Briefly, the three most vascularized areas detected by endoglin were initially selected (so-called hot spots) under ×40 field. Then, microvessels were counted in each of these areas under a 400× field using a Zeiss microscope (Carl Zeiss Microscopy Ltd., Cambridge, UK) by two independent observers who were blinded to the clinico-pathological data; the mean value of three 400× field (0.30 mm^2^) counts was converted into the mean number of microvessels/mm^2^ and recorded as the MVD of the section for the statistical analyses. For each case, the mean diameter (μm) of the endoglin-stained vessels was also calculated and noted. In addition, the microvessel maturation fraction, *i.e.*, the percentage of endoglin-stained vessels that were also immunoreactive for SMA, was assessed by the comparison of the consecutive sections, stained with endoglin and SMA antibodies, respectively. The vessels that were covered in more than 50% of their circumference by pericyte marker-positive cells were defined as a single positive vessel for pericytes, as previously suggested [41,42].

Spearman’s correlation test was carried out to investigate the correlation between the MVD counts and the maturation fraction of the endoglin-stained microvessels and that between MVD counts and the mean diameter of endoglin-positive vessels. In addition, we used the Mann-Whitney test in order to analyze whether the MVD counts or maturation fraction differed in BM derived from diverse primary tumors and to assess whether any difference in their diameter was present between mature and immature vessels.

Follow-up data, including the overall survival (months) of the patients, could be retrieved from our Tumor Registries in 58/78 (74%) cases. The date of surgery for BM was considered as the entry data, while the end point was the length of survival to death for the primary tumor.

The Mantel-Cox log rank test was applied to assess the strength of association between survival time and the MVD or vessel maturation fraction of the tumors. In the analysis, the optimal cut-off point for MVD was derived from the receiver operating characteristic (ROC) curves for discriminating alive from dead patients. With reference to the survival analysis according to the vessel maturation fraction, cases were subdivided into two groups on the basis of the presence of immature vessels (absent *vs.* present).

For the statistical analyses, a probability (*p*) value less than 0.05 was considered statistically significant. Data were analyzed using the SPSS package version 6.1.3 (SPSS Inc., Chicago, IL, USA).

## 5. Conclusions

In conclusion, our findings seem to indicate that the neo-angiogenesis and vascular phenotype widely differ in BM, which may have different dependencies on neo-angiogenesis according to their origin and histotype. This could have striking importance from a practical viewpoint, since tumors with low neo-angiogenesis and mature vascular phenotype may not be adequate candidates to conventional anti-angiogenic therapies. Thus, we suggest caution in the use of anti-angiogenic therapies on patients with BM, as they may develop life-threatening complications with no benefit. On the other hand, as endoglin has been recently proposed as a target for novel anti-cancer treatments [52,53], the evidence of a high number of endoglin-positive vessels in BM may also open novel therapeutic perspectives for the treatment of these tumors. The present study has several limits. Indeed, our findings need to be validated in larger cohorts of patients and the vascular phenotype assessed by additional markers for pericytes, such as NG2. However, the main limit is the lack of correlation of our observations with the clinical response to anti-angiogenic treatments. Thus, further studies solving this issue are warranted to verify if endoglin-MVD and the vessel maturation fraction may represent biomarkers predictive of their efficacy in BM.

## Figures and Tables

**Table 1.** Clinico-pathological characteristics, microvessel density (MVD) counts, vessel diameter and phenotype of the 78 brain metastases (BM) in the study. CRC, colorectal carcinoma; CCRCC, clear cell renal cell carcinoma; MPNST, malignant peripheral nerve sheath tumor.

Case	Age	Sex	Site	MVD (v/mm^2^)	Counts range (v/HPF)	Mean diameter of endoglin-stained vessels (μm)	Maturation index	Primary tumor	Status	FU
1	63	M	parietal	10	3	39.12	1	CRC	Dead	7
2	65	M	parietal	18.8	2–8	25.09	1	CRC	Dead	56
3	58	M	frontal	93.3	25–31	44.17	1	CRC	Dead	5
4	71	M	frontal	46.6	14–16	38.7	1	CRC	Alive	18
5	62	F	frontal	42.2	9–15	22.99	1	breast ductal adenocarcinoma	Dead	43
6	55	F	temporal	80	24	29.13	0.97	breast ductal adenocarcinoma	Dead	17
7	71	F	parietal	20	3–9	39.29	1	breast ductal adenocarcinoma	Dead	43
8	62	F	cerebellar	36	9–12	32.14	1	breast ductal adenocarcinoma	Dead	6
9	40	F	temporal	17.7	4–7	22.17	1	breast ductal adenocarcinoma	Alive	98
10	47	F	temporal	12.2	1–6	25.78	1	breast ductal adenocarcinoma	Dead	4
11	73	F	parietal	40	12	33.97	1	breast ductal adenocarcinoma	n.a.	
12	44	F	parietal	90	24–25	22.68	0.94	breast ductal adenocarcinoma	Dead	6
13	69	F	cerebellar	9.3	2–3	22.52	1	breast ductal adenocarcinoma	Dead	3
14	75	F	parietal	1.11	0–1	74.85	1	breast ductal adenocarcinoma	Alive	27
15	71	F	parietal	70.66	20–22	24.81	0.82	breast ductal adenocarcinoma	Dead	26
16	46	F	cerebellar	58	15–18	26.14	0.84	breast ductal adenocarcinoma	Dead	3
17	60	F	frontal	35.5	9–12	31.18	1	breast ductal adenocarcinoma	Dead	1
18	59	M	cerebellar	1.11	0–1	27.16	1	CCRCC	Alive	8
19	61	M	cerebellar	40	11–12	30.93	1	CCRCC	n.a.	
20	71	F	cerebellar	23.3	5–8	35.19	1	CCRCC	n.a.	
21	57	F	temporal	20	4–8	12.53	1	CCRCC	Dead	33
22	78	M	parietal	54	15–17	42.11	1	thymic carcinoma	n.a.	
23	73	F	temporal	16.6	4–5	47.38	1	melanoma	Dead	24
24	44	M	occipital	57.3	9–19	46.22	1	melanoma	n.a.	
25	68	F	frontal	44	12–14	48.32	1	melanoma	Alive	9
26	69	M	frontal	10	2–4	42.22	1	melanoma	Alive	6
27	58	M	frontal	2.22	0–2	45.28	1	melanoma	Alive	1
28	46	M	frontal	36.66	9–13	43.18	1	melanoma	Alive	3
29	67	F	cerebellar	24.44	4–12	38.17	1	melanoma	Dead	6
30	76	M	cerebellar	15.55	3–6	32.16	1	melanoma	Dead	22
31	66	M	frontal	27.7	7–9	40.11	1	MPNST	Alive	11
32	49	F	frontal	40	6–18	58.17	1	serous papillary ovarian carcinoma	n.a.	
33	80	F	temporo-parietal	93.3	23–31	35.67	1	serous papillary ovarian carcinoma	Alive	13
34	61	F	temporo-parietal	11.3	2–5	29.6	1	serous papillary ovarian carcinoma	Alive	23
35	58	F	parieto-occipital	12	2–7	31.34	1	serous papillary ovarian carcinoma	Alive	59
36	55	F	frontal	43.3	9–15	38.14	1	serous papillary ovarian carcinoma	Alive	21
37	74	M	frontal	97.7	28–30	38.78	0.89	small cell lung carcinoma	Dead	8
38	71	F	cerebellar	38	8–14	39.01	1	lung adenocarcinoma	n.a.	
39	67	M	cerebellar	34.6	8–13	40.35	0.73	lung adenocarcinoma	Dead	1
40	58	M	frontal	100	29–31	39.15	0.96	lung adenocarcinoma	n.a.	
41	59	M	frontal	45.3	10–17	31.25	1	lung adenocarcinoma	Dead	25
42	69	F	temporal	41.3	12–13	48.56	1	lung adenocarcinoma	n.a.	
43	77	M	cerebellar	88.6	19–25	48.92	0.73	lung adenocarcinoma	Alive	35
44	60	M	frontal	28	6–10	24.45	1	lung adenocarcinoma	Alive	20
45	55	F	cerebellar	64.6	16–21	37.06	0.56	lung adenocarcinoma	Dead	2
46	74	M	frontal	33.3	3–15	38.12	1	lung adenocarcinoma	Alive	38
47	50	F	frontal	56.6	15–19	43.13	1	lung adenocarcinoma	Alive	34
48	72	M	cerebellar	140	40–44	41.78	0.59	lung adenocarcinoma	Alive	33
49	65	M	temporal	98.8	27–32	38.78	0.82	lung adenocarcinoma	Alive	24
50	79	F	frontal	41.11	11–13	34.67	1	lung adenocarcinoma	Alive	7
51	46	M	frontal	77.7	19–27	30.78	0.6	lung adenocarcinoma	Alive	68
52	67	M	temporo-parietal	72.2	20–22	32.16	0.86	lung adenocarcinoma	Dead	23
53	73	M	temporal	34.6	10–11	41.12	1	lung adenocarcinoma	Dead	35
54	57	F	parietal	46.6	13–14	29.6	0.72	lung adenocarcinoma	n.a.	
55	61	M	frontal	63.3	15–23	32.78	1	lung adenocarcinoma	Dead	4
56	58	M	parietal	91.1	27–28	45.12	0.8	lung adenocarcinoma	Dead	1
57	69	M	frontal	83.3	24–26	39.37	0.8	lung adenocarcinoma	Alive	4
58	71	M	temporal	6	0–3	26.08	1	lung adenocarcinoma	Dead	19
59	67	F	frontal	12	3–5	38.76	1	lung adenocarcinoma	n.a.	
60	56	M	frontal	100	29–32	21.91	0.83	lung adenocarcinoma	Dead	13
61	49	F	parietal	26	6–10	58.23	1	lung adenocarcinoma	Alive	16
62	60	M	temporal	57.3	19–21	29.58	1	lung adenocarcinoma	Dead	12
63	66	M	frontal	32.6	8–10	38.5	1	lung adenocarcinoma	n.a.	
64	59	M	frontal	72	17–24	59.23	1	lung adenocarcinoma	n.a.	
65	49	M	temporal	12	2–5	42.22	1	lung squamous cell carcinoma	Dead	25
66	50	M	frontal	20.6	4–8	41.11	1	lung squamous cell carcinoma	Alive	16
67	56	M	cerebellar	53.3	4–6	44.35	1	lung squamous cell carcinoma	Alive	17
68	59	M	frontal	13.3	2–5	32.67	1	lung squamous cell carcinoma	n.a.	
69	58	M	temporal	25.5	6–8	40.13	1	lung squamous cell carcinoma	Dead	13
70	57	M	frontal	22.2	5–7	38.14	1	lung squamous cell carcinoma	Dead	4
71	74	M	frontal	83.3	23–27	35.67	0.92	lung large cell carcinoma	Dead	7
72	68	M	cerebellar	123.3	34–38	33.84	0.52	lung large cell carcinoma	Dead	1
73	69	M	temporal	110.6	35–38	29.16	0.8	lung large cell carcinoma	Dead	1
74	72	M	cerebellar	82	23–27	37.94	0.96	lung large cell carcinoma	n.a.	
75	76	M	frontal	66.6	19–21	36.64	0.78	lung large cell carcinoma	n.a.	
76	68	F	parietal	9.3	0–4	37.56	1	uterine clear cell adenocarcinoma	n.a.	
77	70	F	frontal	113.3	30–37	52.34	0.7	uterine endometrioid carcinoma	n.a.	
78	75	F	frontal	4	0–2	35.33	1	uterine serous carcinoma	n.a.	

MVD, microvessel density; V, vessel; FU, follow-up; M, male; F, female; n.a., not available.

**Table 2.** Mean MVD and maturation index according to the origin and histotype of BM.

Primary tumor	Mean MVD (v/mm^2^)	Mean maturation index
lung carcinoma	58.85 ± 33.84	0.89 ± 0.14
*large cell*	93.16 ± 23.12	0.79 ± 0.17
*adenocarcinoma*	58.7 ± 31.49	0.88 ± 0.14
*squamous cell carcinoma*	24.48 ± 15.05	1
*breast ductal adenocarcinoma*	39.43 ± 28.17	0.96 ± 0.06
CRC	42.17 ± 37.48	1
*NOS*	69.95 ± 33.02	1
*mucinous*	14.4 ± 6.2	1
CCRCC	20.82 ± 16.41	1
melanoma	25.84 ± 15.1	1
ovarian serous papillary carcinoma	39.98 ± 33.38	1
uterine carcinoma	42.2 ± 61.63	0.9 ± 0.17
*serous*	4	
*endometrioid*	113.3	
*clear cell*	9.3	

MVD, microvessel density; V, vessel; NOS, not otherwise specified.
